# Study of the Langat virus RNA-dependent RNA polymerase through homology modeling

**DOI:** 10.14806/ej.26.1.944

**Published:** 2021-06-29

**Authors:** Kalliopi Io Diakou, Thanasis Mitsis, Katerina Pierouli, Eleni Papakonstantinou, Vasileios Megalooikonomou, Aspasia Efthimiadou, Dimitrios Vlachakis

**Affiliations:** 1 Laboratory of Genetics, Department of Biotechnology, School of Applied Biology and Biotechnology, Agricultural University of Athens, Athens, Greece; 2 Computer Engineering and Informatics Department, School of Engineering, University of Patras, Patras, Greece; 3 Department of Soil Science of Athens, Institute of Soil and Water Resources, Hellenic Agricultural Organization - Demeter, Attica, Greece; 4 Lab of Molecular Endocrinology, Center of Clinical, Experimental Surgery and Translational Research, Biomedical Research Foundation of the Academy of Athens, Athens, Greece; 5 Department of Informatics, Faculty of Natural and Mathematical Sciences, King's College London, London, United Kingdom

## Abstract

Langat virus is a member of the *Flaviviridae* family and a close relative of a group of important tick-borne viruses that cause human encephalitis. RNA-dependent RNA polymerase is a significant component of the replication mechanism of the *Flaviviridae* viral family. In the present work, a three-dimensional model of the Langat virus RNA-dependent RNA polymerase was designed through homology modeling. The experimentally determined structure of the RNA-dependent RNA polymerase of Dengue virus type II, another member of the same viral family, was employed as template for the homology modeling process. The resulting model underwent a series of optimisations and its quality was verified using the Verify3D algorithm. Important functional characteristics of the family of viral RNA-dependent RNA polymerases were identified in the generated model, thus affirming the potential for its use in the possible design of anti-viral agents for Langat virus.

## Introduction

The genome of the *Flaviviridae* viral family is a single-stranded, non-segmented RNA, usually 9.5–12.3 kilobases long. It contains a large open reading frame (ORF), with untranslated regions (UTRs) at the 5’ and 3’ ends. The 3’ end of the RNA molecule is non-polyadenylated, while the 5’ region presents variability among the various genera (Dimmock *et al.*, 2016). The open reading frame codes for a polyprotein, which is subsequently cleaved by viral and cellular proteases in specific positions, resulting in roughly ten to twelve structural and non-structural proteins. The structural proteins are located in the N-terminal part of the polyprotein, while the non-structural (NS) proteins are located in the C-terminal part (Vlachakis *et al.*, 2012). Structural proteins play a key role in the formation of the viral capsid and envelope, while the non-structural proteins participate in the replication of the viral genome in the cytoplasm (Choi and Rossmann, 2009).

One of the non-structural proteins produced by the cleavage of the polyprotein is NS5, which contains two domains, the N-terminal methyltransferase domain, and the C-terminal RNA-dependent RNA polymerase (RdRp) domain (Murray *et al.*, 2008). RdRp is a critical component of the replication complex, along with helicase and other viral and cellular proteins (Vlachakis and Kossida, 2013; Vlachakis *et al.*, 2013a). During viral replication, the complex first synthesises the complementary strand of the positive-sense RNA strand, creating a double-stranded RNA intermediate. The negative-sense strand of this double-stranded RNA then serves as the template for the synthesis of a new positive-sense RNA strand, which in turn can function as an mRNA molecule for the production of viral proteins, and as genetic material to be packaged into newly-formed virions (Dimmock *et al.*, 2016).

The conformation of viral RdRps resembles a right hand, with subdomains of “fingers”, “palm” and “thumb” that internally form a channel for binding of the template molecule (Wu *et al.*, 2015; Papageorgiou *et al.*, 2016). The RdRp of the flavivirus genus follows the same architecture, with the three mentioned subdomains and a priming loop in the thumb subdomain that is speculated to play a role in making certain that de novo initiation is correct (Duan *et al.*, 2019). The subdomains include seven structural motifs, motifs A to G, that assume key roles in the enzyme’s function, including binding of nucleoside triphosphates (NTPs) and catalysis (Choi and Rossmann 2009; Venkataraman *et al.*, 2018).

Langat virus is a flavivirus and a member of the Tick-borne Encephalitis Virus serocomplex (TBEV serocomplex), along with several other important pathogens that cause human disease, such as Tick-borne Encephalitis virus, Powassan virus and Omsk hemorrhagic fever virus (Gritsun *et al.*, 2003). There have been no recorded cases of human disease caused by Langat virus, a fact that has previously led to the experimental usage of the virus in attenuated vaccines against the more virulent members of the aforementioned serocomplex. However, studies involving the inoculation of lab mice with Langat strains as well as preliminary studies in primates and human volunteers, showed a considerable rate of neurological disease post vaccination (1:10.000 vaccinations) (Seamer and Randles, 1967; Gritsun *et al.*, 2003; Rumyantsev *et al.*, 2006; Maffioli *et al.*, 2014). These findings possibly indicate a potential risk for Langat virus to acquire pathogenic status concerning humans. In this mindset, insights into the molecular mechanisms of the virus’ replication can prove useful towards the understanding of the virus’ life cycle, as well as towards the possible future development of anti-viral agents (Vlachakis, 2009; Vlachakis *et al.*, 2013b). So far, the crystal structure of the RNA-dependent RNA polymerase of Langat virus has not been determined. Hence, in the present work, the three-dimensional (3D) model of the Langat virus RNA-dependent RNA polymerase was designed using a homology modeling approach, by employing the experimentally determined crystal structure of the RNA-dependent RNA polymerase of a virus of the same family, Dengue virus type II (DENV2), as template. The construction of such model can offer a reliable basis for the potential design of anti-viral agents, such as inhibitors (Papageorgiou *et al.*, 2014), in the hypothetical scenario that the virus exhibits capabilities of infecting humans.

## Methods

### Sequence analysis

The amino acid sequence of Langat virus NS5 was retrieved from GenBank database (accession no.: NP_740302.1, entry name: nonstructural protein NS5 [Langat virus]). Protein-protein BLAST algorithm (Altschul *et al.*, 1997) was used through NCBI (Benson *et al.*, 2017) to search for similar protein sequences, using RCSB as the search set. Dengue virus type II NS5 was highlighted as the most suitable to be used as a template. More detailed sequence alignment between the two proteins was carried out using CLUSTALW (Thompson *et al.*, 1994), while the visualisation of conserved residues and RdRp motifs present in the two amino acid sequences was performed with GeneDoc (Nicholas and Nicholas 1997). Secondary structure prediction was carried out using the NPS server (Combet *et al.*, 2000), to ascertain that the alignment would allow the continuation of the process to the next step of homology modeling.

### Homology modeling

The 3D modeling of the Langat virus RdRp was carried out using MOE (Molecular Operating Environment, version: 2016.0801) and the incorporated structure-based homology modeling module (Santiago *et al.*, 2008). The crystal structure of DENV 2 NS5, chain A (PDB entry: 4V0R), was used as the template for the homology modeling process (Zhao *et al.*,2015). Homology modeling in MOE adheres to the following general outline. Regions of the template are copied to assign an initial partial geometry for the sequence. Subsequently, residues with unassigned backbone coordinates are modeled. Lastly, loops are chosen, and side chains are modeled, producing a set of independent models, out of which the best scoring one is selected as the final model. Evaluation of the generated model’s stereochemistry is carried out by the incorporated “Protein Geometry” function.

### Model optimisation

The produced model was further refined with the energy minimisation tool in MOE, using CHARMM27 force field and a dielectric constant of 4 until the conjugate gradient was less than 10–5 kJ/(mol Å), to remove geometrical strain. Additional evaluation of the model’s quality was carried out using Verify3D. This method investigates the similarity of a 3D model with its own amino acid sequence. Considering the environment of every residue, an assortment of known, reliable structures is used to assign a score to each of the twenty amino acids, resulting in a 1D-3D profile (Eisenberg *et al.*, 1997). Finally, the produced model of the Langat virus RdRp was superimposed on the 3D structure of the DENV2 RdRp template to explore similarities and differences between them in terms of secondary and tertiary structure elements, as well as in terms of the respective residues in the amino acid sequences.

## Results

**Figure 1.**
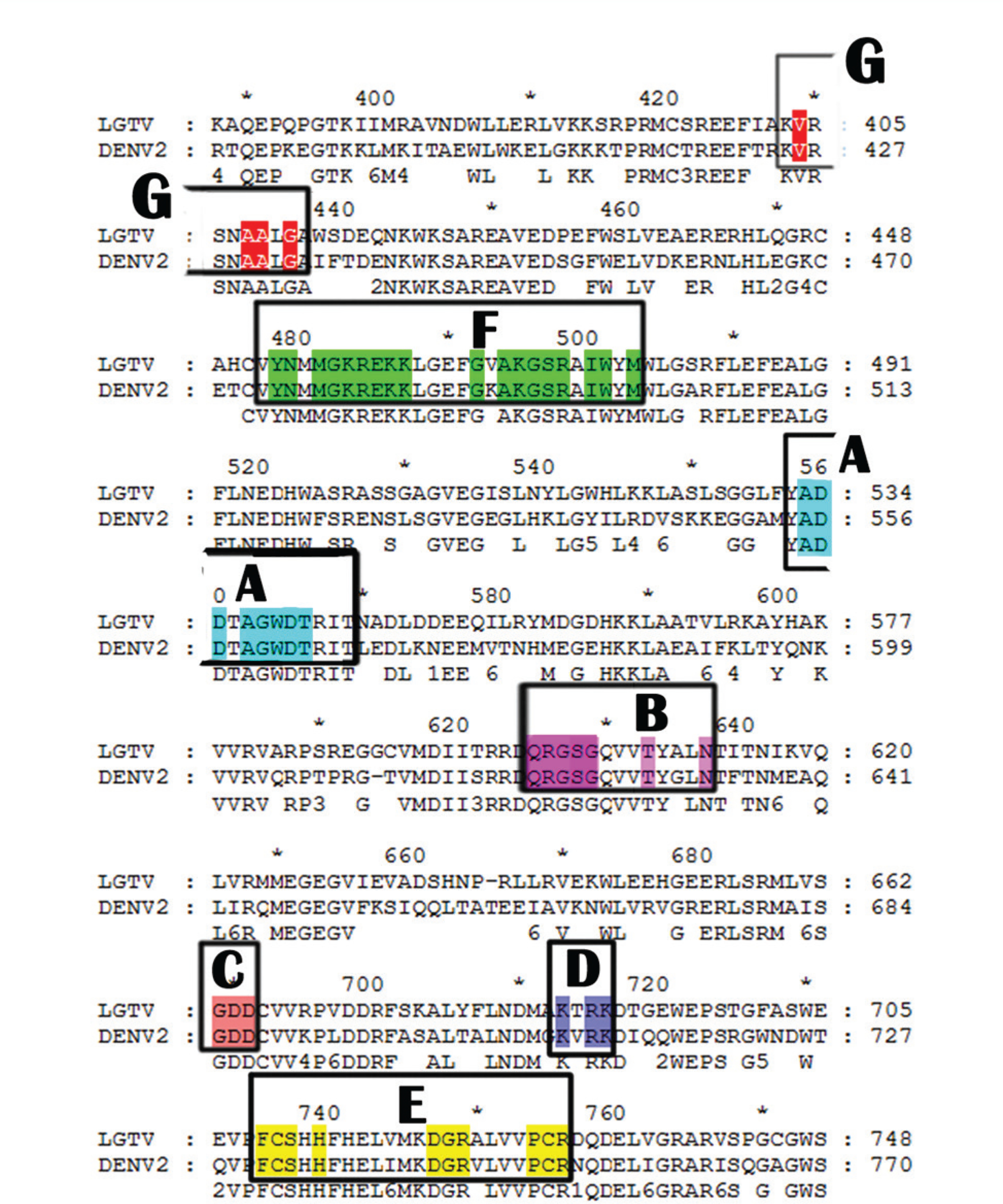
Sequence alignment of the Langat virus (LGTV) RdRp and Dengue virus type II (DENV2) RdRp (entirety of alignment not depicted). The seven major motifs (AG) of *Flaviviridae* RdRps are highlighted.

The amino acid sequences of Langat virus NS5 and DENV2 NS5 were first aligned using the protein-protein BLAST algorithm, resulting in a query cover value of 98% and a percent identity value of 58.8%. Subsequently, CLUSTALW was used for a more detailed alignment of the two sequences and all known RdRp motifs were found to be conserved in the amino acid sequence of Langat virus NS5 (Figure 1).

**Figure 2.**
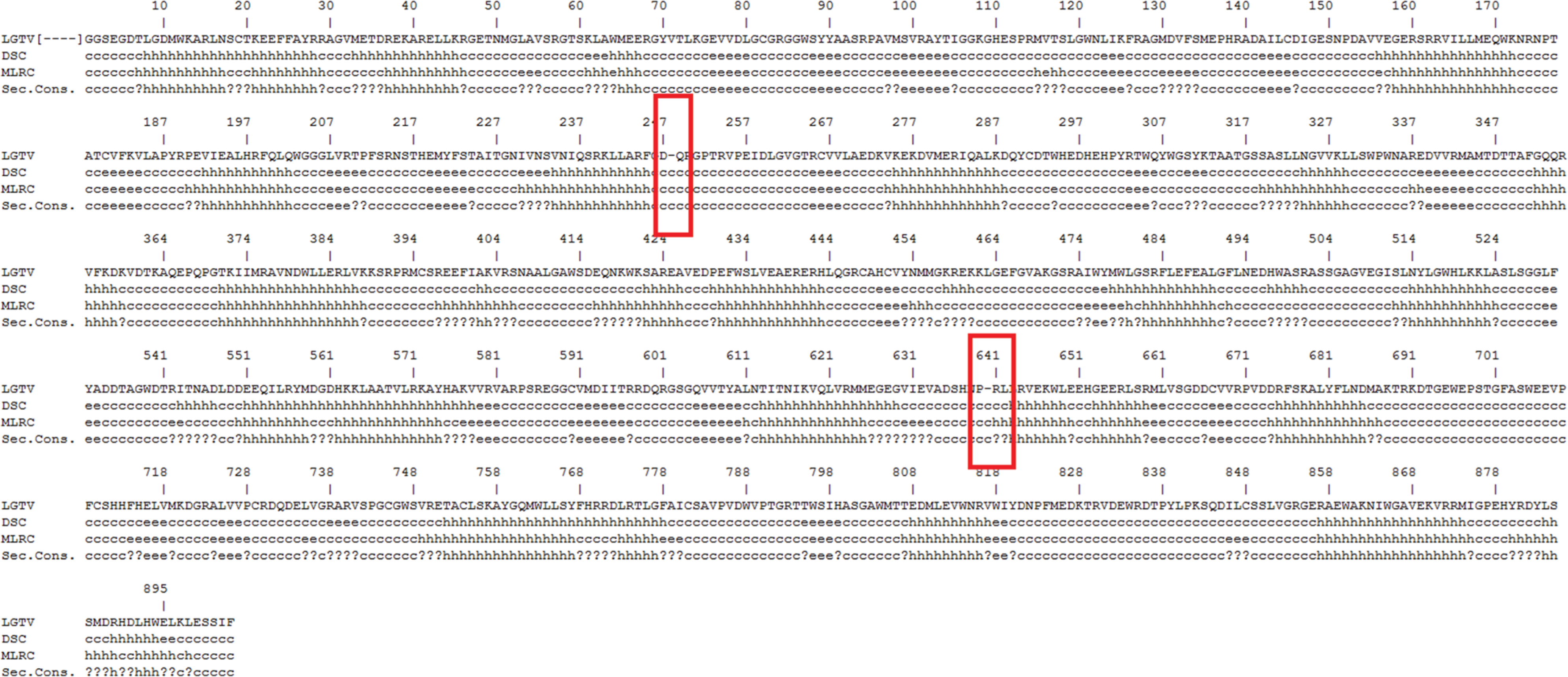
Collective representation of the amino acid sequence of the Langat virus RdRp, including gaps that had been inserted during alignment with DENV2 RdRp. Below them are the predicted secondary structural elements (c: coil, h: helix, e: strand, and (?): ambiguous state are depicted). The areas with inserted gaps and the respective secondary structural elements are denoted in a red box.

Since sequence alignments are unable to take secondary structure elements into account, it was important to evaluate the quality of our alignment and the gaps that the alignment program may have inserted during the alignment. Towards that goal, the NPS server was used to generate a prediction of the secondary structure elements of the Langat virus NS5. As shown in Figure 2, areas of the target sequence with insertion of gaps during the alignment did not correspond to areas where secondary elements such as an alpha-helix or a beta-sheet had been predicted. Hence, these secondary elements appeared intact, ensuring the alignment was correct and allowing a homology modeling approach.

The DENV2 RdRp has been experimentally determined by X-ray crystallography at 2.3Å. Secondary structure prediction performed on the Langat virus RdRp revealed significant similarity to the corresponding structural elements of the DENV2 RdRp. Following the completion of the homology modeling process, the produced model was superimposed to the template, exhibiting an RMSD value of 0.86Å, and was subsequently evaluated within MOE and the incorporated “Protein Geometry” module. The Verify3D algorithm was subsequently used on the model, resulting in scores that ranged from +0.2 to +0.7, confirming the model’s quality, given the fact that low-quality models present scoring below +0.1 (Dym *et al.*, 2001).

**Figure 3.**
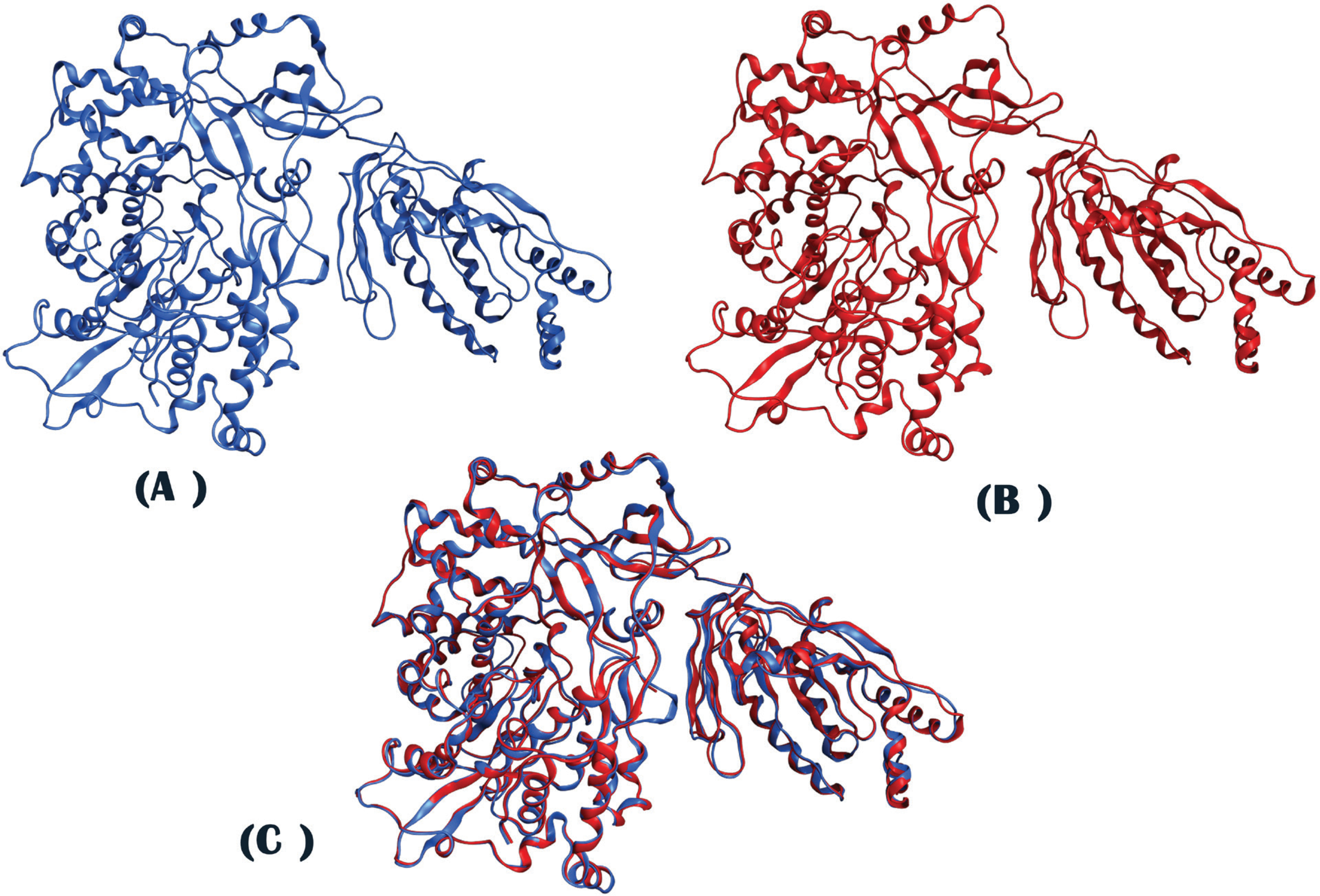
**(A):** Ribbon representation of the generated Langat virus RdRp model in MOE. **(B):** Ribbon representation of the experimentally determined DENV2 RdRp. **(C):** Superposition of the two 3D structures (Blue ribbon: Langat virus RdRp, Red ribbon: DENV2 RdRp).

The generated model of Langat virus RdRp possessed the characteristic structural features of viral RdRps, including the described subdomains of “palm”, “fingers” and “thumb”, as shown by the superposition of the model and the template (Figure 3). Furthermore, motifs of critical importance to the function of viral RdRps, as explained above, were observed to be conserved on a structural level in the produced model.

## Discussion

Non-structural protein 5 (NS5) is the most conserved protein across the flavivirus genus, and its RNA-dependent RNA polymerase domain is of paramount importance for the successful replication of viruses in the *Flaviviridae* family (Bollati *et al.*, 2010). Whereas the RdRp structures of many members of the family, such as Zika virus or Dengue virus, have been experimentally determined, there is an absence of a structure for Langat virus. To bridge this gap, the three-dimensional model of the Langat virus RNA-dependent RNA polymerase enzyme was designed through a homology modeling approach, with the DENV2 RNA-dependent RNA-polymerase used as template. The produced model was found to meet the standards of stereochemical evaluations, ensuring that there were no aberrations in terms of stereochemical characteristics such as bond angles, hydrogen atoms etc. Upon inspection, the model of the Langat RdRp was found to exhibit the key structural characteristics of viral RNA-dependent RNA polymerase enzymes. The conservation of structural characteristics, such as the subdomains, is of great importance for the construction of a reliable enzyme model. The catalytic function of the viral RdRps relies on the correct conformation of the molecule in the three-dimensional space, with experimentally observed changes in activity in the case of alterations of secondary and tertiary structural elements (Malet *et al.*, 2008). The generated model was additionally examined on the level of the RdRp motifs, another essential component of the enzyme activity. The seven motifs that have been described for the RNA-dependent RNA-polymerases, which were conserved on the amino acid sequence of the Langat virus, were also found to be structurally conserved in the model. In summary, the homology modeling process has yielded a three-dimensional model of the Langat virus RdRp enzyme in line with the structural features described for the enzyme family, as well as the *Flaviviridae* family. The generated model remains under the limitations that come with the theoretical nature of the homology modeling process. Nevertheless, the produced three-dimensional model of the Langat virus RdRp can serve as a reliable basis for the exploration of the enzyme’s functions and the virus’ life cycle, until a structure becomes available through an experimental approach.

## Conclusion

Viral infections with members of the *Flaviviridae* viral family as the root cause continue to pose a threat to human health on a global scale. Viruses in the Tick-borne Encephalitis virus serocomplex are responsible for a rising number of cases around the world, a fact that calls for attention from the scientific and medical community. Langat virus, a member of the serocomplex, is a pathogen which while thus far naturally attenuated when it comes to humans, cannot be excluded as a potential future threat. From that perspective, the study of Langat virus’ life cycle is an essential step, in order to be proactive and prepared in case of such outcome. Replication mechanisms are at the core of the virus’ life and transmission, which is why the availability of a structure of the Langat virus RNA-dependent RNA polymerase is of great significance. In the absence of an experimentally determined structure, the 3D model of the enzyme designed through homology modeling can facilitate the analysis of its structure and function in a reliable manner. The extraction of this kind of information can pave the way for further research approaches. For example, the model of the Langat virus RdRp may assist in the rational design of anti-viral agents. These agents, such as potential inhibitors, can be employed in the possible event that Langat virus manifests the ability to cause disease in the human population, reinforcing the level of preparedness of the medical and scientific community.

## Key Points

- Langat virus is serologically related to flaviviruses that cause severe human disease.
- Homology modeling techniques can be used to create a model of the 3D structure of the Langat virus RNA-dependent RNA polymerase, in the absence of an experimentally resolved structure.
- Motifs critical for the enzyme’s functions are present in the resulting model, a significant factor in the construction of a reliable model. An accurate model can be of potential use in the design of anti-viral agents against Langat virus.
